# Interviewing Asylum-Seeking Children: A Scoping Review of Research to Inform Best Practices

**DOI:** 10.1177/15248380241260014

**Published:** 2024-07-24

**Authors:** Shayla Chilliak, Sabrina Musacchio, Tina Montreuil, Shanna Williams

**Affiliations:** 1 McGill University, QC, Canada

**Keywords:** forensic interviewing, war, cultural contexts, memory and trauma

## Abstract

Immigration interviews with asylum-seeking youth have been largely understudied. In domestic legal settings, children interviewed about abuse and maltreatment provide more detailed, relevant responses when asked open-ended questions and when interviewed in a neutral environment, among other supportive practices. In asylum settings, guidance for interviews with youth derives from the United Nations Convention on the Rights of the Child. It is not clear to what extent best practices are employed during asylum interviews with youth. This scoping review was performed to (a) provide an overview of empirical literature on interviews with children in immigration settings, including border screenings, interviews with representatives, and asylum hearings, (b) explore whether best practices derived from forensic psychology and children’s rights are observed in asylum interviews, (c) identify unique interview needs of asylum-seeking youth, and (d) derive implications for research and practice. A scoping review of three databases conducted in October 2023 yielded titles, of which 29 articles met inclusion criteria. These comprised quantitative and qualitative studies in English from 2003 to 2023. Three articles identified were quantitative, and 26 were qualitative. While several articles touched on interview practices and youth’s experiences of interviews, only a few examined how asylum-seeking youth responded to different interview factors such as question type and interview setting. Key findings highlight inconsistent application of best practice principles, and several areas where best practices to support asylum-seeking children require clarification through further research.

Across diverse international contexts, children’s asylum decisions rest on whether a child would risk persecution, torture, death, or mistreatment if returned to their home country (Reisdorf, 2021). This decision may require children to report past experiences of trauma and maltreatment during border screenings, interviews with legal representatives, and immigration hearings, where officials seek information about why the child migrated and what dangers they would face if returned (Warren & York, 2014). Notably, a rising number of youth are migrating without a parent or caregiver (United Nations High Commissioner for Refugees [UNHCR], 2009); these youth are termed “unaccompanied,” and are more likely than other youth to be interviewed upon arrival and during asylum application processes in their host country.

The migration process is often dangerous and asylum-seeking children are at a high risk of undergoing varied traumatic experiences, both prior to and during migration; these include family separation, natural disasters, and witnessing or experiencing assault, torture, human trafficking, slavery, and war (Jensen et al., 2013). Rates of post-traumatic stress disorder among asylum-seeking youth have ranged from 19% to 54% across samples (Bronstein & Montgomery, 2011; Blackmore et al., 2020), with higher rates among unaccompanied children (Given-Wilson et al., 2016). Mental health difficulties such as depression, anxiety, and sleep difficulties have also been identified in children seeking asylum (Blackmore et al., 2020; Montgomery, 2011). Of note, factors related to the asylum process itself may contribute to worse mental health outcomes for these youth (Hornfeck, 2022), underlining the importance of ensuring that asylum processes are conducted with intention and care.

An important variable in this process that can impact both child well-being and outcomes of asylum claims is how children are interviewed by officials. Due to developmental limitations in memory, language, and social understanding, asylum-seeking children “may not be able to articulate their claims to refugee status in the same way as adults and, therefore, may require special assistance to do so” (UNHCR, 2008). Forensic psychology research on children’s domestic legal testimony provides many insights into children’s abilities to remember and report the traumatic events they have experienced in interview and courtroom settings, making this research highly relevant to asylum interviews with children. Indeed, the majority of research on children’s disclosure of experiences of violence, abuse, and maltreatment has been conducted under the purview of forensic psychology (see Lavoie et al., 2021; Saywitz et al., 2016). Research in this field has identified that children are highly susceptible to be influenced by leading and suggestive questioning (Peterson et al., 1999; Waterman et al., 2001), forced-choice (i.e., yes/no) questions (Peterson & Grant, 2001), interviewer pressure or demeanor (Baker et al., 2021; Benedan et al., 2018; Vagni et al., 2015) and other interview-specific issues (i.e., time related questions, McWilliams et al., 2023; ground rules questions, Henderson et al., 2022; McWilliams, Williams et al., 2021; McWilliams et al., 2023). Several child-friendly practices are thus recommended to support children’s accurate reporting during interviews; such guidelines are formulated in dialog with forensic researchers, whose findings inform recommended practices and who in turn study whether these practices are applied in courtroom and forensic interviews (Bruer et al., 2021; Saywitz & Camparo, 2009). For instance, the interview should be conducted in a neutral setting and should begin with a child-friendly opening statement, rapport-building, and narrative practice, in which children recall a neutral event (Lyon et al., 2019; McWilliams, Stolzenberg et al., 2021). Interviewers should use facilitators, supportive statements, and open-ended questions (Bruer et al., 2021, 2022; Feltis et al., 2010). It is recommended that children be given a choice in the gender of their interviewer, particularly when sexual abuse is suspected (American Professional Society on the Abuse of Children, 2012). Objective recordkeeping, such as audio or preferably video recording, also is recommended.

Asylum interviews present many similar challenges to children’s domestic legal testimony. Children may be interviewed by officials or during asylum hearings about experiences such as trauma, maltreatment, or witnessing violence, which they may find it difficult to recall and describe. Recommended practices within immigration contexts largely derive from the United Nations Convention on the Rights of the Child, the global consensus on safeguarding children’s rights and welfare (UN General Assembly, 1989). Several articles of the Convention on the Rights of the Child pertain to children’s rights in asylum processes. Under Article 22, states are obliged to offer protection and humanitarian aid to youth seeking refugee status, while Article 2, the principle of non-discrimination, states that children will not be subject to discrimination based on individual, social, and political factors, and should thus be protected when fleeing discrimination. Article 3 presents the best interests of the child principle, stating that “the best interests of the child shall be a primary consideration” in all actions concerning children (UN General Assembly, 1989). Further, Article 12 presents a child’s right to participate and express their views during legal processes pertaining to themselves.

The UNHCR provides additional guidance on the interpretation and application of the best interests of the child principle to asylum interviews (2008). Namely, a comprehensive assessment should be conducted to determine a child’s best interests, taking into consideration the child’s cultural and familial background, reasons for leaving home, and fears of return. Each child should be interviewed by an official who, along with interpreters, has relevant training in issues of gender, age, and cultural sensitivity. Interviews should be informal, use accessible language, and take place in a confidential and child-friendly location, preferably chosen by the child. Interviews should be recorded, with protocols and notes retained in a file. To support the child’s participation, the interviewer should ensure they understand the asylum process and should support them in sharing their views. To avoid repeated interviews, the interviewer should collect information and present it directly to decision-makers. Caution should be exercised when interviewing children in front of parents or guardians, as this may influence children’s responses. Decisions on a child’s best interests should be made by a panel rather than a single member, should include the child’s views, and should consider that trauma and developmental stage may lead to incomplete or inaccurate recollections from children.

Several maltreatment and trauma-related factors impacting children’s interview responses may be exacerbated in asylum-seeking children due to the potential frequency, severity, and duration of traumatic experiences (Quas & Lyon, 2019). For instance, children are often reluctant to share information out of fear of harm to themselves or their family members (Alaggia et al., 2019; Lev-Wiesel et al., 2014), a concern which may be particularly salient for youth whose family members remain in their country of origin. Of note, best practice guidelines for interviewing asylum-seeking youth have been proposed based on research in domestic legal settings (Quas & Lyon, 2019). However, the experiences of young asylum-seekers may also be unique in ways that impact their interview needs and behaviour. Child refugees experience context-specific stressors including pre-migration trauma, in-transit separation from family and parents, and post-migration stressors, such as uncertain legal status (Bean et al., 2007; Ellis et al., 2008; Heptinstall et al., 2004). In the context of asylum interviews, research suggests that child refugees may avoid discussing past experiences (van Os et al., 2020) due to fear of flashbacks, nightmares, and intrusive thoughts (Vickers, 2005), and may view secrecy as a protective strategy to maintain agency and control (Chase, 2010). Asylum-seeking children may also mistrust representatives, interpreters, and lawyers due to negative prior experiences with authorities in their home countries (De Haene et al., 2010; Majumder et al., 2015). Interpretation, language, and cultural sensitivity have also been highlighted as important considerations in interviews with asylum-seeking youth (UNHCR, 2008). Of note, the above research on factors impacting asylum-seeking youth largely derives from research in the contexts of medical, social work, and psychotherapy interviews, rather than immigration interviews specifically. While UNHCR guidelines are available to inform asylum interview administration, it is unclear to what extent these are implemented.

The present review aims to identify empirical literature on interviews with children in immigration settings (i.e., border screenings, interviews with representatives, and asylum hearings). Of particular interest is what interview practices are employed with asylum-seeking youth, whether best practices from forensic psychology and UNHCR are upheld during immigration interviews, how youth experience such interviews, and whether asylum-seeking youth require different interview considerations than children in domestic legal settings.

## Method

**Figure 1.**
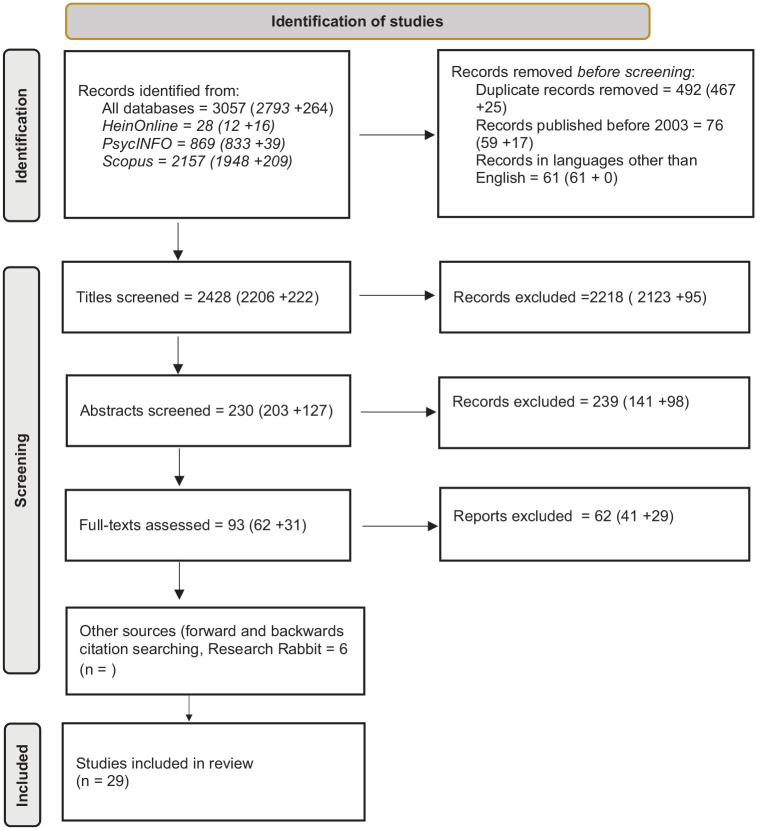
PRISMA flow diagram for search strategy and article screening. *Note.* Numbers in brackets with “+” indicate titles found during the first and second search, respectively, as described in Table 1.

A scoping review was conducted in line with Joanna Briggs Institute guidelines (Peters et al., 2020). In advance of our search, a protocol was drafted and preregistered at https://osf.io/8kzgf/ to ensure transparency (Peters et al., 2022). Preliminary search strategies for the PsycINFO and HeinOnline databases were reviewed by academic librarians in Education and Counseling Psychology and Law, respectively. The PsycINFO search strategy was refined for the Scopus database. The database search, conducted in October 2023, is outlined in Table 1. EndNote software was used to compile results and remove duplicates. Titles, abstracts, and full texts were then screened for adherence to inclusion and exclusion criteria by two reviewers using Rayyan, a tool for conducting systematic and scoping reviews (www.rayyan.ai). After the full-text review, a second search was conducted with new search terms identified during the first search. The second round of titles was screened through identical steps to the first. Reference lists of all included articles, and those of excluded literature reviews on adjacent topics, were backward-screened by the first author. Included publications were forward-screened using Google Scholar’s “cited by.” Lastly, included articles were entered into online literature-mapping tool Research Rabbit, and thematically related titles were also screened. A Preferred Reporting Items for Systematic Reviews and Meta-Analyses (PRISMA) diagram detailing the steps taken in this review is presented in Figure 1.

**Table 1.** Scoping Review Search Strategy.

Search Stage	Database	Search Fields	Search Terms
First search	PsycINFO	Keyword search	*Search one*: (exp interviews/OR exp interrogation/OR exp questioning/OR exp interviewing/OR exp suggestibility/) AND (exp asylum seeking/ OR exp political asylum/OR exp refugees)
		Title and abstract search	((asylum OR refugee OR unaccompanied) AND (youth OR child* OR kid* OR underage OR minor OR minors OR adolescent*) AND (interview* OR interrogat* OR questioning)).ti.ab.
	Scopus	Title, abstract, and keyword search	((asylum OR refugee OR unaccompanied) AND (youth OR child* OR minor OR minors) AND (interview* OR interrogat* OR questioning)).ti.ab.
	Hein Online	Title search	(child* OR minor OR minors OR youth OR young OR adolescen* OR teen*) AND (refugee OR migrant* OR immigrant* OR asylum OR unaccompanied OR separated OR immigration) AND (interview* OR questioning OR hearing OR testimony OR determination)
Second search	PsycINFO	Keyword search	(“best interests of the child” OR “Convention on the Rights of the Child” OR “child-centered”) AND (asylum OR “asylum seek*” OR refugee* OR unaccompanied)
	Scopus	Title, abstract, and keyword search	(“best interests of the child” OR “Convention on the Rights of the Child” OR “child-centered”) AND (asylum OR “asylum seek*” OR refugee* OR unaccompanied)
	Hein Online	Title search	(“best interests of the child” OR “Convention on the Rights of the Child” OR “child-centered”) AND (asylum OR “asylum seek*” OR refugee* OR unaccompanied)

Studies were included if the research questions or study findings pertained to interviews with asylum-seeking during border screenings, immigration hearings, and with lawyers or representatives. Interviews within health, psychiatric, and other contexts were excluded. Experimental studies examining asylum-seeking youths’ interview responses were included. Studies of young asylum-seekers’ migration narratives, post-migration experiences, and determinants of well-being were retained until the abstract or full-text review stage and were included if results addressed asylum interviews. Studies were included in which participants were asylum-seekers under 18 or stakeholders (i.e., legal representatives, NGO workers), in which adults retrospectively discussed seeking asylum as children, and which analyzed interview notes, transcripts, and audio recordings. Qualitative and quantitative studies published in English-language peer-reviewed journals from 2003 to 2023 were included. Conference proceedings, reviews, practice guides, gray literature, protocols, validation studies, interviews, legal commentaries, and methodological papers were excluded. Key findings from included articles were extracted based on pertinence to the scoping review research questions; these were tracked by the first author in a spreadsheet in Microsoft Excel, refined into Table 2.

**Table 2.** Scoping Review Results and Key Findings.

Article	Sample	Study Design/Methodology	Interview Context	Key Findings
Ballucci and Ghebrai (2023) *Canada*	*N* = 4 case files Unaccompanied minors’ asylum decisions (ages 15–17, 2 female)	Qualitative Critical discourse analysis of asylum files (transcripts, statements, and decisions)	Asylum hearing	Children were seen as untrustworthy; interviewers challenged children’s honesty. Interviews took place in a formal setting. Impacts of trauma on testimony were not considered. Officials saw less “child-like” youth as less credible.
Bryan and Denov (2011) *Canada*	*N* = 34 Unaccompanied asylum-seeking youth (*n* = 17, ages 17–30, 3 female), and stakeholders (*n* = *17*, 12 female)	Qualitative Analysis of retrospective report interviews with youth and stakeholders	Border crossing and asylum hearing (judges, prosecutors)	Immigration officials and prosecutors questioned youths’ honesty and used argumentative questioning. Youth were seen as complicit in being smuggled. Some youth saw race and class as influencing their treatment. Youth experienced disrespectful comments regarding their physical appearance.
Chase (2010) *United Kingdom*	*N* = 54 Unaccompanied asylum-seeking youth (ages 9–18)	Qualitative Thematic analysis of retrospective report interviews	Not specified—migration experience	Barriers to youth testimony included confusion and fear, as some had been warned in transit not to be open and honest. Some youths were not provided interpretation for interviews. Youth reported fear and feeling dehumanized.
Childs et al. (2021) *United Kingdom*	*N* = 31 Unaccompanied asylum-seeking youth (*m_age_* = 18.15, *n* = 13) and age-matched peers (*m_age_* = 17.53, *n* = 18)	Quantitative Experimental comparison of suggestibility of asylum-seeking youth vs. controls, and influence of past exposure to violence	Lab experiment	Responses of separated youth (versus controls) were more susceptible to change due to interviewer pressure. Experience of negative life events was associated with greater vulnerability to interrogative pressure.
Connolly (2015) *United Kingdom*	*N* = 29 Unaccompanied asylum-seeking youth	Qualitative Thematic analysis of retrospective report interviews	Police stations, border crossings, and immigration interviews	Interviews with police were described as hostile, but interpreters helped youth feel less fearful. Border screenings were not conducted separate from accompanying adults, facilitating trafficking. During Home Office interviews, youth reported being disbelieved and made fun of, and ignored when requesting rest and medical attention. Expression of reasons for seeking asylum was restricted by standardized interview questions.
Crawley (2010) *United Kingdom*	*N* = 27 Unaccompanied asylum-seeking youth	Qualitative Analysis of interviews with youth and ethnographic observation in an Asylum Screening Unit	Asylum interview	Youth felt that interviewers were suspicious, lacked empathy, and did not explain the interview’s purpose. Interview questions were described as close-ended and unclear, and interviewers were uninformed about youths’ countries of origin.
Doering-White (2018) *Mexico*	*N* = 23 Unaccompanied asylum-seeking youth (*n* = 8, ages 15–19, 4 female) and practitioners (*n* = 15)	Qualitative Ethnographic observation in migrant shelters and analysis of interviews with youth and service providers.	Border crossing, screening interviews, and interviews with support staff for asylum applications	Association with gangs or smugglers disqualified youth from asylum as they were not considered vulnerable. Immigration agents pressured youths to sign documents consenting to deportation. Support staff who interviewed youth focused on providing care and asking youths’ reasons for migrating. Youth were mistrustful of support staff, even those helping with asylum applications.
Dursun and Sauer (2021) *Austria*	*N* = 29 Unaccompanied asylum-seeking youth (*n* = 12, ages 14–23, 1 female) and stakeholders (*n* = 17)	Qualitative Analysis of retrospective report interviews with youth and stakeholders	Entire migration experience	Participants expressed fear of police. They reported being treated with suspicion by authorities, which made them hesitant to provide information. Youth experiences were deemed irrelevant and not solicited/permitted during asylum hearings.
Galli (2018) *United States*	*N* = 30 Unaccompanied asylum-seeking youth (*n* = 22, ages 12–18) and asylum-seeking adults (*n* = 8)	Qualitative Ethnographic observation of legal interviews with asylum-seeking youth	Interview with legal representatives	Youth had difficulty recounting events and explaining political context in their country of origin. Including other adults in interviews can provide information but can also silence youth. Representatives used suggestive questioning to probe for fear of return, and coached youths’ stories, vocabulary, self-presentation (i.e., dress, eye contact), and emotional expression (i.e., crying) before asylum interviews. Youth were advised to downplay agency and emphasize victimization during hearings.
Gornik (2022a) *Slovenia*	*N* = 19 Unaccompanied asylum-seeking youth	Qualitative Individual and group interviews with youth and ethnographic observation of an asylum group home	Border crossing and asylum hearing	Youth noted insufficient time, support, and information before asylum hearings. Hearings were long (3–6 hr). Youth reported anxiety before and during hearings and wished for social support. Trust-building and interpretation during interview were inadequate. Questions were not age- or culture-appropriate. Youths’ statements during border crossing, without an interpreter, were used to challenge their credibility by finding inconsistencies in their accounts.
Gornik (2022b) *Slovenia*	*N* = 19 Unaccompanied asylum-seeking youth (ages 13–17, 0 female)	Qualitative Individual and group interviews with youth and ethnographic observation of an asylum group home	Border crossing and asylum hearing	Participants lacked understanding of asylum procedures and felt powerless. Trust-building and interpretation were deemed lacking. Hearings were too long (3–6). Interviews were not adapted to age, education, or cultural background. Youth statements were scrutinized for inconsistencies, compared with their untranslated border entry statements, and focused on small details.
Hedlund (2017) *Sweden*	*N* = 916 cases Unaccompanied minors’ asylum decisions (84.72% male)	Qualitative Thematic analysis of migration case officers’ case decisions	Asylum hearing	Case officers used argumentative techniques and expected coherent, rational, and informed answers about youths’ country of origin. Inability to provide information was interpreted as lack of credibility.
Huynh (2021) *United States*	*N* = 12 juvenile dockets *N* = 26 stakeholders	Qualitative Grounded theory analysis of ethnographic observation of immigration court hearings and interviews with stakeholders	Asylum hearings	Judges allocated little time per case and heard multiple cases together. Hearings were long, seating uncomfortable, and food and drink not permitted. Legal representatives met children only minutes before the hearing. Interpretation quality was poor (i.e., provided via phone, with omitted portions). Children were asked only factual information (i.e., age, name, country of origin). Due to timeline, lawyers could not substantiate children’s fear of return with research or family members’ testimony.
Jain and Lee (2018) *United States*	*N* = 5 cases Notes from Credible Fear Interviews with asylum-seeking minors	Qualitative Critical case sampling analysis of case notes: five cases resulting in negative asylum decisions	Credible fear interview	Interviews lacked a child-friendly opening statement and rapport-building, used of adversarial questioning, failed to investigate children’s expressions of fear of harm, and lacked sensitivity to trauma. Officials did not ask if children wanted to speak without a parent in the room. Interviews were not recorded.
Keselman et al. (2008) *Sweden*	*N* = 26 Russian asylum-seeking minors (13–18 years, *m_age_* = 16.1 years, 6 female)	Quantitative Analysis of interview transcripts for question type and interpretation accuracy	Screening and asylum interview (immigration case workers and interpreters)	Interviewers used more close-ended (43%) than open-ended (37%) questions. Suggestive questions were infrequent (2%). Children’s behavior was criticized during interviews (5%). Trained interviewers used more open-ended questions. Thirty-Three percent of statements were modified during interpretation. Open-ended questions were more frequently translated correctly.
Keselman, Cederborg, Linell et al. (2010) *Sweden*	*N* = 26 Russian asylum-seeking minors (14–18 years, *m_age_* = 16.0 years, 6 female)	Qualitative Discourse analysis of interpreter“side-sequences” during interviews	Screening and asylum interviews (immigration case workers and interpreters)	Six of 18 interpreters used side-sequences, which excluded children, distorted their voices, and influenced their responses. Interpreters also violated principles of neutrality during interviews by trying to coach children and by challenging the truth of their statements.
Keselman, Cederborg, Lamb et al. (2010) *Sweden*	*N* = 26 Russian asylum-seeking minors (14–18 years, *m_age_* = 16.0 years, 6 female)	Quantitative Analysis of impact of question type on disclosure, and mistranslation frequency	Screening and asylum interviews (immigration case workers and interpreters)	Open-ended question types yielded relevant responses more often than suggestive or option-posing questions. Sixteen percent of interpreter renditions were inaccurate.
Linell and Keselman (2011) *Sweden*	*N* = 26 Russian asylum-seeking minors (14–18 years, *m_age_* = 16.0 years, 6 female)	Qualitative Analysis of distrust in asylum interview transcripts	Screening and asylum interview	Analyses indicate the structure and content of interviews contribute to mistrust. Mistrust escalates through sequences of mistrusting exchanges. Focus on discrepant details increases mistrust.
Lundberg (2011) *Sweden*	*n* = 105 case records of children’s asylum decisions *n* = 35 handling officers interviewed	Qualitative Analysis of asylum files and interviews with handling officers	Asylum interview	Children were not offered the opportunity to speak nor interviewed individually. Children’s statements were not weighed heavily in decisions. Best interest principles were mostly used to justify negative decisions. Youth participation was limited by officials’ mistrust, fear of retraumatizing children, lack of officials’ confidence in their own competency, and time constraints.
Mellinger (2022) *United States*	*N* = 28 Immigration lawyers	Qualitative Analysis of interviews with immigration lawyers about Asylum Office hearings	Asylum Office Interviews	Interpretation quality was inconsistent, depending on which officer was assigned to the case, or youth were required to bring their own interpreter. Unaccompanied youth were guaranteed an interpreter only if they did not have an attorney. Poor interpretation was perceived to anger Asylum Officers and to endanger clients’ asylum claims.
Munro et al. (2013) Canada	*N* = 40 Newcoming LGBT youth (*n* = 39, ages 14–29) and service providers (*n* = 1)	Qualitative Thematic analysis of group and individual interviews with youth and stakeholders	Not specified- migration experience	For refugee claims based on fear of persecution due to sexuality, youth felt they were expected to fit stereotypes of gay appearance, dress, and comport, and felt they had to “prove” their sexuality. Judges challenged individuals not fitting stereotypes. Black participants felt their sexuality was particularly doubted.
Ottosson and Lundberg (2013) *Sweden*	*N* = 9 Lawyers acting as children’s advocates	Qualitative Analysis of child advocate reports of interview strategies used with asylum-seeking children and families	Interview with legal representative	Representatives often did not include children in the process, particularly young children. Children’s grounds for asylum were not considered separately from parents’. Representatives justified exclusion based on children’s vulnerability and inability to testify, and by stating that children did not have independent asylum claims. Some children were hesitant to share information in front of their parents, however, children’s unique asylum claims may improve chances of a positive decision.
Pearce (2011) *United Kingdom*	*N* = *72* Practitioners *N* = *37* case study record reviews	Qualitative Focus groups and individual interviews with practitioners (social workers, NGO workers, border and police agents); archival analysis of cases		Youth, and especially boys, were often hesitant to disclose trafficking experiences, while adults often disbelieved accounts when youth did disclose. Participants lacked clarity on the definition of trafficking, with some believing youth can consent to being trafficked. Some port-of-entry interviews disregarded youths’ explicit reports of trafficking.
Rap (2022) *Netherlands*	*N* = 21 Asylum-seeking children, accompanied and unaccompanied (ages 12–22, 12 female)	Qualitative Retrospective interviews with youth regarding their rights to be informed and participate in asylum hearings	Asylum interview and hearing	Children were stressed and apprehensive before interviews. Some hearings were conducted without lawyers or representatives. Interviews in front of family members caused youth discomfort sharing information. Youth reported inaccurate interpretation, the same question asked several times, not understanding the purpose of very detailed questions, and stressed at not knowing answers.
Rap (2023a) *Netherlands*	*N* = 42 Professionals working with refugee children	Qualitative Analysis of professionals’ views on children’s participation in asylum procedures.	Interviews with legal representatives and asylum hearing	Lawyers emphasized the need to give detailed accounts and used visual supports. Immigration officials’ explained the interview purpose, established rapport, adapted language, and explained the purpose of questions. Children were often not joined by lawyers or representatives. Accompanied children were not interviewed.
Rap (2023b) *Netherlands*	*N* = 13 Unaccompanied asylum-seeking children (ages 7–11, 4 female)	Ethnographic observation of asylum interviews with youth	Asylum interviews	A child-friendly interview room was provided and interviews were recorded. Toys and visual aids were available. Interview procedure, purpose, and ground rules were often neglected. Rapport-building was used by some interviewers. Children were often asked for specific details and comprehension of language was not verified. Interviewers relied on close ended and “why did. . .” questions which may be difficult for children.
Schmidt (2022) *United States*	*N* = 77 Unaccompanied asylum-seeking children from Mexico and Central America (ages 12–17, 35 female)	Qualitative Secondary analysis of interviews with unaccompanied youth	Interviews with researchers about reasons for migration	Less than half of youth disclosed maltreatment in response to questions about reasons for migration. Most disclosures followed questions about living situations or school. Most youth did not see maltreatment as motivating their migration; however, their experiences nonetheless should inform asylum decisions.
Shamseldin (2012) *England, Ireland, & Sweden*	*N* = 59 Professionals working with unaccompanied youth	Qualitative Analysis of stakeholder interviews on implementation of Convention on the Rights of the Child	Interviews with professionals in social services, immigration, and NGOs	Children’s right to participate in asylum proceedings was recognized but was only considered to apply to older children. Younger children were seen as unable to participate. Interpretation of “best interests” differed between participants.
Torres et al. (2022) *United States and Mexico*	*N* = 51 Unaccompanied Mexican and Central American youth (*n* = 25, ages 12–17, >50% female), and service providers (*n* = 26)	Qualitative Ethnographic observation, questionnaires, and interviews with youth at Mexican migrant shelters near the US border	Border screening interviews	Youth were not asked if they feared return prior to deportation and were provided no hearing or representative, compromising due process. Mexican youth reported verbal and physical abuse by border officials, being pressured to sign voluntary return papers in English, and being threatened with prolonged detention as coercion. Many youths were deported to unsafe circumstances.

## Results

The scoping review yielded 29 articles that met inclusion criteria, comprising a total of 872 participants and 1,067 archival records (see Table 2). A total of 499 asylum-seeking youth participants were included across all studies, the majority of whom were unaccompanied. The articles described research conducted in Sweden (*n* = 8), Canada (*n* = 3), the United States (*n* = 6), the United Kingdom (*n* = 6), Mexico (*n* = 2), the Netherlands (*n* = 4), Slovenia (*n* = 2), Ireland (*n* = 1), and Austria (*n* = 1). Results included three quantitative and 26 qualitative studies. Of the qualitative studies, eight analyzed archival materials and records (i.e., asylum decisions, interview notes, transcripts of asylum interviews, and observation of juvenile court dockets, *n* = 1,067), 12 analyzed individual and group interviews with asylum-seekers, 10 analyzed interviews with stakeholders (i.e., advocates and lawyers; *n* = 347), and eight analyzed ethnographic observations of youth shelters, courtrooms, and legal offices. Several studies triangulated data from multiple sources.

The phenomena of interest for this review included interview practices (i.e., questioning techniques used during interviews), interview experiences (i.e., how youth experienced, interpreted, and felt about the interview), and interview responses (i.e., how youth responded to questions during interviews). Of the included studies, only three directly examined how youth respond during asylum interviews (Childs et al., 2021; Keselman, Cederborg, Lamb et al., 2010; Schmidt, 2022). In contrast, almost all articles touched on interview practices; most of these were qualitative studies in which youth and stakeholders discussed interview practices they had witnessed or experienced. Thus, there was considerable overlap between articles on interview practices and interview experiences, as youth reported both how interviews were conducted and how they felt about these experiences. Only eight studies examined interviews directly. Of these, three reported on direct ethnographic observations of court rooms and lawyers’ offices (Galli, 2018; Huynh, 2021; Rap, 2023b) and five analyzed transcribed recordings of interviews (Ballucci & Ghebrai, 2023; Keselman et al., 2008; Keselman, Cederborg, Linell et al., 2010; Keselman, Cederborg, Lamb, et al., 2010; Linell & Keselman, 2011).

A source of bias in this review stems from the fact that four of seven identified articles examining interview practices use the same dataset (Keselman et al., 2008; Keselman, Cederborg, Linell et al., 2010; Keselman, Cederborg, Lamb et al., 2010; Linell & Keselman, 2011); these findings should thus be interpreted with caution. In addition, the two studies by Gornik (2022a, 2022b) use the same dataset, same methodology, and have highly similar research questions.

### Summary of Key Findings (summarized in Table 3)

**Table 3.** Critical Findings.

Of 29 articles identified on immigration interviews with asylum-seeking youth, 26 were qualitative and three quantitative, highlighting the dearth of quantitative research on this topic.
Only three articles studied how youth respond during interviews based on variations in interview practices.
Only eight articles directly studied interviews via recordings, transcripts, or ethnographic observation.
Forensic best practices for interviewing children were frequently neglected: • Rapport-building, explanation of the interview’s purpose, and a child-friendly opening statement were often absent. • Interviews often took place in overly formal spaces (lawyers’ offices or courtrooms), and were an inappropriately long duration. • Interviews used close-ended questions, yielding less information and increasing likelihood of inaccurate responses due to suggestibility • Some interviewers used argumentative questioning techniques • Translation was often of inadequate quality.
Impacts of trauma on children’s memory and testimony were not consistently considered. • Several studies found children distrusted interviewers, exacerbated by antagonism of officials, and by children’s prior experiences. • Children often do not feel comfortable discussing traumatic events in front of their parents or caregivers, who may be unaware of such events (UNHCR, 2008). It is therefore important that youth be provided the opportunity for an interview separate from parents. • Some studies found that interviewers did not consider the impact of trauma on children’s ability to describe past events, interpreting knowledge gaps and inconsistencies as indicating lack of credibility.
Both accompanied and unaccompanied youth frequently felt excluded from participation in asylum processes, in violation of the CRC article 12 ensuring children’s participation and voice in asylum processes. • Immigration officials felt unprepared to interview youth directly, and fears of inadvertently retraumatizing youth motivated their exclusion from asylum processes. • It was assumed children’s claims were identical to their parents, and parents were de fact assigned to represent their children, thus excluding children.

#### Rapport, Information, and Setting

Youth reported high anxiety and stress before and during asylum hearings, highlighting the need for child-friendly practices to build rapport and trust (Chase, 2010; Gornik, 2022a; Rap, 2022). Two qualitative studies found that immigration officials used positive practices, such as introducing the interview procedure, explaining the interview’s purpose, establishing rapport, adapting language, taking frequent breaks, and explaining the purpose of questions (Rap, 2023a, 2023b). Contrarily, other qualitative studies found that child-friendly practices were neglected, such as explaining the interview’s purpose (Crawley, 2010; Huynh, 2021; Rap, 2023b), establishing trust prior to the interview, and providing sufficient time, legal advice, and information (Gornik, 2022b; Huynh, 2021). As a result, youth reported not understanding the asylum process, which resulted in feelings of powerlessness (Gornik, 2022b). Analyses of case files indicated neglect of child-friendly opening statements and interview settings (Ballucci & Ghebrai, 2023; Jain & Lee, 2018) and long duration of hearings (Gornik, 2022a; Huyhn, 2021). One study found that interviews were not recorded, limiting accountability (Jain & Lee, 2018). Ethnographic observation of lawyers, on the other hand, found that representatives did provide information to prepare youth for asylum hearings. Lawyers used strategies to support their clients, including emphasizing the need to provide true and detailed accounts and using visual supports to help youth understand the asylum process and timeline (Rap, 2023a).

#### Interpretation

Several studies identified problematic interpretation practices. Poor quality interpretation was noted by youth (Chase, 2010; Gornik, 2022a; Rap, 2022), stakeholders (Mellinger, 2022), and ethnographic observation (Huyhn, 2021). Studies by Keselman et al. (2008), Keselman, Cederborg, Lamb et al. (2010), Keselman, Cederborg, Linell et al. (2010) found that 33% of statements from either interviewer or interviewee were modified by interpreters. Side-sequences between interpreters and either party excluded children from interview participation, distorted children’s statements, and influenced their responses (Keselman, Cederborg, Linell et al., 2010). Concerningly, two US-based studies found lack of interpretation during children’s asylum hearings (Huynh, 2021; Mellinger, 2022).

#### Question Type

Several studies identified inappropriate questioning. Qualitatively, stakeholders described that questions were not adapted to children’s age, education, or cultural background (Gornik, 2022b). Youth described being asked close-ended and unclear questions (Crawley, 2010), and that standardized questions limited their ability to describe their experiences (Connolly, 2015). Ethnographic observations of asylum interviews also noted predominance of close-ended and “why” questions, which children may struggle to answer due to limitations in cause-and-effect reasoning. Quantitatively, interviewers used more close-ended (43%) than open-ended (37%) questions; trained interviewers asked more open-ended questions, which were translated correctly and yielded more relevant responses than close-ended questions (Keselman et al., 2008; Keselman, Cederborg, Lamb et al., 2010).

Legal representatives also used both close-ended and suggestive questions (Galli, 2018). Because youth often have difficulty recounting past events and explaining the political context of their home country, representatives used strategies such as including other adults in interviews and using suggestive questioning to establish a fear of return. They also coached youths’ stories, vocabulary, self-presentation (i.e., dress, eye contact), and emotional expression prior to hearings (Galli, 2018). However, youth providing migration narratives in a research context were more likely to disclose maltreatment in response to broad questions about school or home, as opposed to direct questions about their reasons for migration, highlighting the utility of open-ended questions to elicit information relevant to asylum claims (Schmidt, 2022).

### Mistrust, Pressure, and Argumentative Questioning

Studies of case files and qualitative interviews with youth and stakeholders found that immigration officials and prosecutors used argumentative questioning styles and challenged children’s honesty during interviews (Ballucci & Ghebrai, 2023; Bryan & Denov, 2011; Hedlund, 2017; Jain & Lee, 2018). Young participants described officials as suspicious and lacking empathy, which in turn increased youths’ reticence (Crawley, 2010; Dursun & Sauer, 2021; Linell & Keselman, 2011). Boys were particularly hesitant to disclose information about being trafficked (Pearce, 2011). Several studies found that inconsistencies in youths’ statements were used by immigration officials to challenge their statements and to argue against their credibility in asylum decisions (Ballucci & Ghebrai, 2023). One study described that statements made during border crossing in the absence of an interpreter were used to invalidate youth’s later testimonies (Gornik, 2022a). Conversely, youth expressed distrust of police and other officials (Dursun & Sauer, 2021); contributing to difficulties identifying child trafficking (Pearce, 2011).

Social pressure was also identified during interviews, comprising 5% of interviewer statements (Keselman et al., 2008). Similarly, youth reported that the same question was sometimes asked several times, causing confusion and distress (Rap, 2022). A qualitative study of youths’ responses to pressure during interviews found that separated asylum-seeking youth were more susceptible to change their interview responses under pressure than controls, a tendency that was further exacerbated by prior experience of violence (Childs et al., 2021).

### Trauma-Sensitive Considerations

Notably, children who have experienced violent or traumatic events may not feel comfortable discussing these events with their parents or caregivers (UNHCR, 2008). However, two studies found that officials did not ask youth if they wanted to be interviewed separately from parents, which in one instance limited disclosure, and in another caused distress to a child interviewed in front of their parent (Jain & Lee, 2018; Rap, 2022). Connolly (2015) found that the failure to separate children from their caregivers during border screenings facilitated trafficking.

Three studies found that children were not asked about their fear of return during border screenings and asylum hearings (Ballucci & Ghebrai, 2023; Jain & Lee, 2018; Torres et al., 2022). Immigration officials further did not consider the impact of trauma on children’s ability to provide a coherent, consistent account of past events. Knowledge gaps and inconsistencies were seen as indicating lack of credibility (Hedlund, 2017). Youth were expected to fit a child-like profile, and youth who appeared more mature were seen as less credible (Ballucci & Ghebrai, 2023). Youth who had been smuggled were also seen as complicit in their smuggling and were barred from seeking asylum during border screenings (Bryan & Denov, 2011; Doering-White, 2018). Similarly, queer youth filing asylum claims based on fear of persecution due to sexuality felt pressure to fit stereotypes to “prove” their sexuality (Munro et al., 2013).

### Application of Best Interest Principles

Lundberg (2011) found that best interest principles were mainly used to justify negative case decisions in the name of family reunification, contrary to UNHCR guidance. Another study highlighted officials’ conflicting views on how this principle should be interpreted (Shamseldin, 2012). Two studies found that young Mexican youth asylum-seekers in the United States were routinely pressured to sign “voluntary removal” documents consenting to be deported, often in English (youths’ first language was Spanish) and under threat of indefinite detention, in clear contravention of their best interests (Doering-White, 2018; Torres et al., 2022).

### Youth Participation

The Convention on the Rights of the Child proscribes youths’ participation in legal processes such as asylum applications. Several studies found that youths’ experiences were not solicited, were deemed irrelevant, or were not considered in asylum decisions (Dursun & Sauer, 2021; Lundberg, 2011; Shamseldin, 2012). Stakeholders believed that children were vulnerable, and unable to testify, and their asylum claims would be identical to those of their parents (Ottosson & Lundberg, 2013). However, parents may not be aware of their children’s unique asylum claims (Ottosson & Lundberg, 2013). Other practices, such as hearing several youths at the same time and conducting rushed hearings, also limited participation in concerning ways (Huynh, 2021).

## Discussion

The current scoping review presents the first systematic collection of empirical research on interviews with asylum-seeking youth in immigration settings. We identified 29 articles published during the last 20 years describing (a) interview practices used by immigration, border, and support personnel, (b) how youth experience asylum interviews, and (c) how asylum-seeking youth respond to interview questions. These findings provide valuable information about current practices that can inform policy development and implementation aimed at ensuring the well-being of asylum-seeking children.

The articles in this review point toward several implications for improvement of current practices when interviewing young asylum-seekers (see Table 4). Across multiple studies, best practices for interviewing children were frequently neglected, with rapport-building, explanation of the interview’s purpose, and narrative practice described as inadequate (Ballucci & Ghebrai, 2023; Crawley, 2010; Gornik, 2022b; Huynh, 2021; Jain & Lee, 2018; Rap, 2023b). It is important that these practices be implemented to avoid confusion about the interview’s purpose and to facilitate children’s trust in interviewers (Majumder et al., 2015; Ní Raghallaigh, 2014).

**Table 4.** Implications for Practice, Policy and Research.

Practice and policy:	Training and oversight are necessary to ensure application of best practices for interviewing children established in forensic psychology are followed across immigration contexts (i.e., border screening and asylum hearings).
	Interviews with asylum-seeking youth should be recorded (audio and ideally video) to facilitate accountability.
	In tandem with the above, immigration-related bodies (i.e., border services and immigration administration) should collaborate to avoid re-interviewing youth.
	Children should be interviewed separately from their parents to allow space to discuss information they wouldn’t share in front of their parents, as children often have unique asylum claims.
	Asylum-seeking youth should be provided access to interpreters trained in neutrality. Oversight of interpretation quality should be conducted regularly.
	State bodies should collaborate with researchers to allow access to de-identified interviews with asylum-seeking youth while protecting youths’ identities and safety. Further research is needed to improve public and scholarly knowledge of current practices.
Future research:	Further research is needed on how lawyers and representatives probe for fears of return during interviews with asylum-seeking youth. Lawyers in our review used leading and suggestive questions to establish a fear of return. However other research supports the utility of open-ended questions in asylum contexts to elicit reasons for migration.
	Future research should examine re-interviewing of youth seeking asylum across contexts (ie., border crossing, lawyers, and legal decision-makers) and the impact of re-interviewing on their interview responses.
	Further quantitative research, for instance on the frequency of question types and utility of different question types in asylum interviews is recommended.

Another area for improving current practices in asylum interviews is language interpretation. Across several studies in our review, quality interpretation services were lacking (Chase, 2010; Gornik, 2022a; Huyhn, 2021; Mellinger, 2022; Rap, 2021); additionally, interpreters overstepped the bounds of their roles, adding information to both child and interviewer statements (Keselman, Cederborg & Linell et al., 2010). As a result, children’s voices were distorted and their participation in the asylum process was compromised. To address this issue, interpreters must receive training in interpreter neutrality and should be provided routine oversight in this regard.

Several review inclusions also highlighted questioning style as an area needing improvement in terms of current practices. Several studies highlighted use of close-ended questions, in contravention of best practice guidelines (Crawley, 2010; Keselman et al., 2008; Rap, 2023b). However, practitioner perspectives on the use of forced-choice questions and suggestive questions sometimes contradicted best practices. Specifically, lawyers and advocates in one study supported suggestive questioning to fill in gaps in youths’ stories and to help them articulate fears of returning to their country of origin (Galli, 2018). Given that a positive asylum decision requires a credible fear of return, suggestive questions were considered necessary to elicit specific information. This practice conflicts, however, with research finding that open-ended questions yield more accurate and detailed information from children (Lyon & Henderson, 2021). Other studies in this review similarly support open-ended questioning, which yielded more relevant responses (Keselman, Cederborg & Linell et al., 2010) and greater maltreatment disclosures (Schmidt, 2022) than close-ended and wh- questions. Therefore, it is recommended that asylum interviews make primary use of open-ended questions about a child’s life prior to migrating, rather than close-ended questions currently in use. How best to establish fears of return without leading questions, perhaps integrating open-ended interviewing with information gathered from outside sources is an important question for future research.

Finally, practice improvements are needed in terms of recordkeeping practices during asylum interviews. Jain and Lee (2018) noted that border screenings are not routinely recorded. This limits accountability, as border agents’ interviews are not subject to review. Further, Pearce (2011) points out the failure of border screenings to identify trafficked youth, even when youth disclose trafficking explicitly. Overall, it is therefore crucial that border agents receive the same training and oversight as other immigration officials, and that their interviews be recorded to ensure accountability.

Some of the above concerns may be addressed by way of states adopting a policy of appointing representatives to conduct the interview process with children in an informal, one-on-one context, as recommended by the UNHCR’s guidance for determining the best interests of the child (2008). In Canada, for instance, unaccompanied youth are assigned a designated representative, allowing the opportunity to build trust prior to discussing their asylum claims (Immigration and Refugee Board of Canada, 2022). Representatives also reduce the likelihood that children will be interviewed multiple times, in line with UNHCR guidance (2008). Such solutions do not absolve other officials, such as border officers and judges, from training and diligence in employing best practices. Another policy that should be implemented across states is a national guideline for consideration of developmental issues in the immigration process, already present in some states (e.g., Immigration and Refugee Board of Canada, 2022). Further, it is important to ensure that when representatives are assigned, adequate training is provided, interpreters are used, and adequate recordkeeping and oversight are provided. Youth with representatives should also be offered the opportunity to participate in asylum hearings, in line with the Convention on the Rights of the Child article on children’s participation.

Studies in this review found that both accompanied and unaccompanied youth frequently felt excluded from participation in asylum processes, in violation of article 12 of the Convention on the Rights of the Child. One reason for this exclusion was that immigration officials felt unprepared to interview youth directly due to fears of inadvertently retraumatizing them, highlighting that immigration officials involved in questioning or interviewing asylum-seekers must be adequately trained and prepared to interview children. Importantly, Connolly (2015) points out that a potential consequence of not interviewing children directly is a failure to identify child trafficking. Additionally, immigration officials often assumed that children’s claims were identical to their parents’, and subsequently failed to interview accompanied children (Dursun & Sauer, 2021; Lundberg, 2011; Ottosson & Lundberg, 2013; Shamseldin, 2012). It is in fact important to modify policy and practice in this regard to implement interviews with youth separate from parents, as youth may not feel comfortable disclosing incidents of violence, threat, or trauma directly to or in front of their parents (UNHCR, 2008). Interviews should thus be conducted with all children seeking asylum, including those accompanied by parents, with appropriate mental health support provided outside of the asylum process.

Concerningly, one study found that the Convention on the Rights of the Child principle ensuring the protection of the best interests of the child was predominantly used to order children’s deportation rather than their resettlement (Lundberg, 2011). The Convention on the Rights of the Child states that protecting children from violence, neglect, exploitation, trafficking, child labor, armed conflict, and underage recruitment to conflict should be prioritized above other factors, such as family unification, in best interest determinations. This particular finding suggests that articles of the Convention on the Rights of the Child are being misinterpreted and misapplied, resulting in threats to children’s rights. This finding underlines the importance that nations develop policies on refugee determination for children which are informed by UNHCR guidance. Articles also highlighted timeline issues in conflict with UNHCR guidance (2008). While the determination process of the child’s best interests is recommended to take place as soon as possible, some studies reported that youth felt they were given inadequate time to prepare for their hearings, which exacerbated stress. While waiting extended periods for asylum decisions can also cause stress (Fazel et al., 2005; Thommessen et al., 2015), a reasonable timeline with both reduced wait time and adequate advance notice is recommended.

### Scoping Review Limitations

This review’s findings should be interpreted in consideration of some limitations. Firstly, included studies were limited to those published in English, thereby excluding literature from outside the English-language publishing sphere. Additionally, multiple analyses of single datasets may have introduced bias into the scoping review results, where the findings emerging from these samples are given inflated importance in our review. Namely, the four studies with Keselman as first and second author (Keselman et al., 2008; Keselman, Cederborg, Linell et al., 2010; Keselman, Cederborg, Lamb et al., 2010; Linell & Keselman, 2011) represent multiple analyses of the same dataset with differing research questions, while the two studies by Gornik (2022a, 2022b) present highly similar research questions and analyses. Third, the included studies had different methodologies, research questions, and study protocols; results are therefore not comparable across studies and should not be taken to fully represent any individual country’s asylum practices. It is also important to note that the findings of included studies do not all emerge from neutral research questions. For instance, while some studies set out to examine value-neutral questions such as how youth experience asylum interviews, others investigated negative phenomena such as homophobia or discrimination. As such, the overall results skew toward identifying the ways in which asylum interviews cause harm or fail to meet established standards, although some studies did emphasize positive practices of professionals and officials.

### Scoping Review Strengths

While previous reviews have been conducted on adjacent topics such as barriers and facilitators of disclosure for refugee children (van Os et al., 2020), elements of the best interests of the child determination (van Os et al., 2016), and autobiographical memory in asylum-seeking adults (Herlihy et al., 2012), this report presents the first review of empirical research on interviewing asylum-seeking youth. Given that asylum-seeking youth are marginalized along multiple axes including language, age, race, class, and citizenship status, our review supports diversity by specifically highlighting youth’s personal experiences of asylum processes, via review and description of qualitative studies which include young asylum-seekers’ experiences and perspectives. Further strengths of this review include preregistration of a review protocol, adherence to recommended practices for scoping reviews, systematic search and screening procedures, and assessment of bias in the review findings. Most importantly, this review provides the first synthesis of research on asylum interviews with children and youth, from which we derive recommendations for the development of policies and practices to safeguard the rights and well-being of asylum-seeking youth moving forwards.

### Scope of Current Research and Future Directions

The literature on interviewing asylum-seeking youth is limited compared to research on interview practices with children in domestic legal contexts (i.e., forensic interviews and children’s legal testimony). A promising direction for future research on asylum interviews with children is to attempt to replicate findings from forensic psychology research, which has examined the effectiveness of interview practices such as rapport-building, narrative practice, backchannelling, and open-ended questions, among others (e.g., Henderson et al., 2022; Lavoie et al., 2022; McWilliams et al., 2021; Sullivan et al., 2022). Based on our review, the vast majority of research on asylum interviews with children is qualitative in nature. While this type of research provides a rich understanding of children’s asylum interviews, it is by definition not suited to provide quantifiable data on the extent of problematic practices. Moreover, several qualitative studies emerged from research questions on adjacent phenomena, such as immigration and post-immigration experiences (Bryan & Denov, 2011; Doering-White, 2018; Dursun & Sauer, 2021; Munro et al., 2013; Torres et al., 2022). Finally, most studies identified in the present review focused on asylum-seeking children who were unaccompanied; future research should examine the experiences of accompanied asylum-seeking youth with regards to asylum interviews, and the migration and asylum application processes more broadly (Bhabha, 2014).

Increased quantitative research in this field is needed to clarify current practices across diverse national contexts and to monitor implementation of best practices as discussed above. Future research in this field should consider the extent of adherence to best practices quantitatively, to determine needs for training and oversight of professionals conducting interviews. Such research, however, requires direct analysis of interviews via case files, recordings, transcripts, or direct observation. Access to such documents is often limited by governments due to concerns around confidentiality, in line with UNHCR guidance. In order to facilitate the study of interview practices with asylum-seeking youth, policy adaptations are needed to support partnerships between state institutions and researchers to remove barriers to accessing data while ensuring protection of youths’ anonymity and safety.
